# Theranostic Contact Lens for Ocular Cystinosis Utilizing Gold Nanoparticles

**DOI:** 10.3390/bios15010016

**Published:** 2025-01-03

**Authors:** Eunbe Ha, Hwajeong Kang, Hyeran Noh

**Affiliations:** 1 Department of Optometry, Seoul National University of Science and Technology, 232 Gongneung-ro, Nowon-gu, Seoul 01811, Republic of Korea; eunbe@seoultech.ac.kr (E.H.); zx3233@seoultech.ac.kr (H.K.); 2 Convergence Institute of Biomedical Engineering and Biomaterials, Seoul National University of Science and Technology, 232 Gongneung-ro, Nowon-gu, Seoul 01811, Republic of Korea

**Keywords:** ocular cystinosis, gold nanoparticles, theranostic, biosensor, contact lens

## Abstract

Ocular cystinosis is a disease in which accumulated cystine crystals cause damage to the eyes, necessitating timely treatment and ongoing monitoring of cystine levels. The current treatment involves frequent administration of cysteamine eye drops, which suffer from low bioavailability and can lead to drug toxicity, making it essential to prescribe an appropriate dosage based on the patient’s condition. Additionally, cystine crystal levels are typically assessed subjectively via slit-lamp examination, requiring frequent clinical visits and causing discomfort for the patient. In this study, we propose a theranostic contact lens that simultaneously performs therapy and diagnosis on a single platform utilizing gold nanoparticles (GNPs). The binding interactions between GNPs and cystine were confirmed in solution, and thermodynamic analysis further elucidated the bonding force between the two substances. With a comprehensive understanding of these interactions, we investigated the potential of the theranostic GNP-loaded contact lens (GNP-CL). Upon exposure to various concentrations of cystine, the GNP-CL demonstrated distinct color changes, transitioning from red to blue. This color shift enabled quantitative monitoring of cystine levels. The treatment efficacy was validated by confirming a reduction in cystine concentration following the reaction. This platform has the potential to improve disease management in ocular cystinosis by reducing the reliance on cysteamine and offering an objective self-monitoring tool that does not require specialized equipment.

## 1. Introduction

Cystinosis is a rare genetic metabolic disorder characterized by the accumulation of cystine crystals in the lysosomes, leading to progressive damage in vital organs, including the eyes, kidneys, liver, and brain [1,2]. This autosomal recessive condition results from mutations in the CTNS gene, which encodes cystinosin, a membrane transport protein responsible for cystine clearance. The dysfunction of cystinosin impedes cystine transport, causing its accumulation and crystallization within lysosomes [3]. Ocular cystinosis, a common manifestation of all forms of cystinosis [4], involves the deposition of needle-like cystine crystals in ocular tissues and tears, leading to symptoms such as stinging, photophobia, blepharospasm, and foreign body sensation [5,6]. If untreated, cystine crystals progressively invade deeper corneal layers, resulting in severe complications, including recurrent erosions, photoreceptor degeneration, papilledema, and eventual blindness [7,8].

The effective management of ocular cystinosis requires early intervention and continuous monitoring. Due to the avascular nature of the cornea, oral medications are ineffective, making cysteamine eye drops the standard treatment. Cysteamine works by reducing cystine levels, converting cystine crystals into a cysteine–cysteamine complex [9]. However, these eye drops have low ocular bioavailability (<5%), necessitating frequent administration—typically every hour—which interferes with daily activities. Moreover, the high frequency of dosing increases the risk of drug overdose or toxicity [10]. Efforts to improve drug bioavailability, such as more viscous formulations and drug-delivery systems, have been explored [11,12]. However, these solutions are still based on cysteamine, which causes a burning sensation upon application and is prone to oxidation, making storage difficult. Therefore, accurately prescribing the right dosage based on the patient’s condition remains critical [13].

Currently, cystine accumulation is assessed subjectively using slit-lamp examinations, where an ophthalmologist scores cystine density on a scale from 0.00 to 3.00, but exact quantification is not possible [7]. This approach, which often requires the use of slit-lamp lights near the cornea, can be uncomfortable for patients, especially those with photophobia. Additionally, patients must undergo periodic visits to ophthalmology clinics for monitoring and follow-up care. This process can be inconvenient and may lead to gaps in treatment, accelerating disease progression and causing more severe complications [8,13].

A theranostic approach—an integrated approach combining therapy and diagnosis—has emerged as a promising tool in disease management, offering personalized treatment, preventing complications, and enabling the real-time assessment of therapeutic efficacy [14]. This field is expanding beyond cancer research [15] to include areas such as dentistry [16] and ophthalmology. In particular, contact lenses have been investigated as a wearable platform for drug delivery and the non-invasive monitoring of ocular conditions [17]. These platforms range from smart contact lenses with integrated chips that require additional devices to simpler systems that monitor disease progression by detecting color changes in the lens [18].

In this study, we propose a theranostic approach utilizing a gold nanoparticle-loaded contact lens (GNP-CL) to both lower the cystine level by removing lenses from the eyes after a reaction with cystine and enable the easy monitoring of cystine levels through color changes in the lens. Among various nanomaterials, we chose gold nanoparticles (GNPs) due to their two key properties, namely (1) their strong affinity for cystine [19,20,21], which enables effective binding and removal, and (2) their ability to undergo colorimetric changes that facilitate non-invasive cystine monitoring [22,23]. Additionally, GNPs possess antibacterial properties, low toxicity, and biocompatibility, making them ideal for drug-delivery systems, diagnostic kits, and theranostic applications [24,25,26]. To assess the theranostic function of GNP-CL, we investigated their performance both in solution and in the contact lens state. This platform offers a simple accessible method for disease management without the need for specialized equipment, providing patients with an effective tool for monitoring ocular cystinosis.

## 2. Materials and Methods

### 2.1. Materials

Gold (III) chloride trihydrate (HAuCl4, 99.9%, 16961-25-4, Mw 393.83) and cystine (L-cystine, 56-89-3, Mw 240.30) were purchased from Sigma Aldrich (Burlingto, MA, USA). Sodium citrate dihydrate (Na3C6H5O7⋅2H2O, 6132-04-3, Mw 294.10) purchased from Daejung Chemicals (Siheung-si, Gyeonggi-do, Republic of Korea) was used without further purification. HCl (HCl 35–37%, 7647-01-0, Mw 36.46) was purchased from Samchun Chemicals (Gangnam-gu, Seoul, Republic of Korea). Formic acid (HCOOH, 64-18-6, Mw 46.08, HPLC grade) and acetonitrile (CH3CN, 75-05-8, Mw 41.05, HPLC grade) were provided by WAKO chemicals (Chuo-Ku, Osaka, Japan) and Daejung Chemicals (Siheung-si, Gyeonggi-do, Republic of Korea), respectively. Non-ionic hydrogel contact lenses (Proclear 1-day soft contact lens) were purchased from Cooper Vision (Juana Diaz, PR, USA). Solutions of 20 mM cystine were prepared as stock solutions by dissolving 0.048 g L-cystine in 0.4 M HCl. All samples of cystine solution were used by diluting stock solutions with distilled water.

### 2.2. Preparation of GNPs

GNPs were synthesized via the Turkevich method [27]. Briefly, 2 mL of sodium citrate (84 mM) was rapidly added to a 90 °C HAuCl4 solution (18 mL, 0.5 mM). This mixture was continuously stirred at a speed of 500 rpm for 30 min, resulting in the formation of a red colloidal solution. After cooling to room temperature (25 °C), the solution was purified using a 5 nm pore-sized cellulose tube and then stored at 4 °C for further use. GNPs were characterized using UV–visible spectroscopy (Nano-MD, Scinco, Gangnam-gu, Seoul, Republic of Korea), Dynamic light scattering (Zetasizer, Malvern, UK), and transmission electron microscopy (Cs-STEM, JEOL, Akishima, Tokyo, Japan). The concentration of GNPs was calculated as 4.8 nM using the previously reported formula [28].

### 2.3. Reaction Between GNPs and Cystine in Solution State

A total of 4.8 nM GNPs and 200 μM cystine were reacted at a 1:1 ratio for 1 h at room temperature. The morphology of the mixture was obtained by Cs-STEM. Then, cystine concentration was increased from 0 to 400 μM in 50 μM increments, and the reaction was carried out under the same conditions. We observed the color change in the mixture and the UV-vis spectra.

In addition, to observe the effect of temperature, the mixture (4.8 nM GNPs and 400 μM cystine, 1:1 ratio) was placed in an incubator set to various temperatures (5, 10, 20, 30, 40, 50 °C), and UV-vis spectra were measured every 2 min for the first 10 min and then every 5 min thereafter.

Isothermal titration calorimetry (ITC) experiments were conducted using the PEAQ-ITC instrument (Malvern Panalytical Ltd., Malvern, UK) at 25 °C. An injection syringe was filled with 40 μL of cystine (1 mM), and 200 μL of GNPs was placed in the sample cell. The reference cell was filled with distilled water. The experimental protocol involved a single 0.4 μL injection followed by 13 injections of 3 μL of the cystine solution into the GNP solution. For the control measurements, 20 mM HCl solution with dissolved cystine was titrated instead of the cystine solution to eliminate any other effects on the GNPs. The data were then fitted using a sequential binding model from the PEAQ-ITC instrument.

### 2.4. Preperation of GNP-Loaded Contact Lens

Prior to loading, to remove the influence of the lens preservative, two contact lenses were soaked in 10 mL of distilled water for 12 h, and this process was repeated twice. Each cleaned lens was immersed in 2 mL of 2.4 nM GNPs solution for 24 h. Then, the moisture content was calculated according to the following formula [29]:

(1) $$\mathrm{Water}\;\mathrm{Content}=\frac{W_{m}-W_{d}}{W_{m}}*100\;\left(\%\right)$$

The contact angle was measured by the sessile drop method [30] and analyzed through ImageJ 1.53e software.

To observe the reaction with cystine, GNP-CLs were submerged in various concentrations of cystine ranging from 0 to 40 μM (1 mL). The reaction was performed in an incubator set to 25 °C for 30 min. Afterward, the GNP-CLs were placed between glass slides and scanned using a printer (DCP-T500W, Brother Industries, Mizuho-ku, Nagoya, Japan). The average color value of the scanned images was quantified using Adobe Photoshop 2024. The quantification equation was fitted as a cubic function using Origin 2024.

### 2.5. Quantitative Analysis of Cystine Removal

The Waters UPLC system (ACQUITY QDA Mass Detector H-Class UPLC, Waters, Milford, MA, USA) equipment was used to quantify the amount of cystine removal. In the aqueous solution state, the GNP concentration was varied (0 to 4.8 nM) and reacted with 100 μM cystine in a 1:1 ratio. In the contact lens state, the cystine concentration was set as a variable, and GNP-CL was immersed in a cystine solution ranging from 0 to 50 μM (1 mL). All experiments were conducted for 1 h at 25 °C and 200 rpm in an incubator. After the reaction, samples were centrifuged twice for 30 min at 13,500 rpm. These supernatants were filtered through a PTFE filter.

The chromatographic separation was achieved using the C18 column (2.1 mm × 50 mm, 1.7 µm, Waters, Gwangmyeong-si, Gyeonggi-do, Republic of Korea) using the same conditions as described in a previous paper [31]. The mobile phase consisted of acetonitrile (A) (containing 0.05% formic acid) and water (B) (containing 0.05% formic acid) at a flow rate of 0.2 mL/min. Isocratic elution was performed as 50% A. Column and autosampler temperatures were maintained at 30 °C and 10 °C, respectively. The injection volume was 2 μL and the total run time was 3 min. The positive ion mode of electrospray ionization (ESI) was chosen for mass spectrometric analyses. The *m/z* value was 241.1, the corresponding optimized cone voltage was 25 V, and the collision energy for the analytes was 0.8 V.

## 3. Results and Discussion

### 3.1. Morphological Analysis of GNPs and GNPs with Cystine

**Figure 1:**
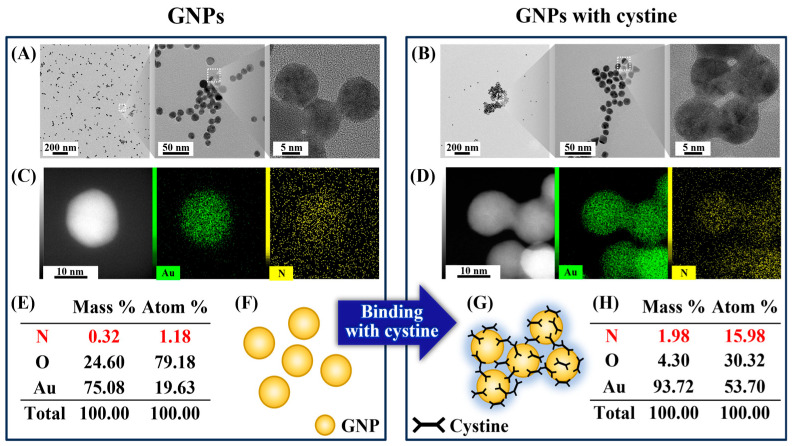
TEM images showing (**A**) GNPs without an amorphous layer and (**B**) GNPs with cystine forming an amorphous layer; (**C**–**E**,**H**) EDS mapping showing nitrogen distribution around the nanoparticles; (**F**,**G**) schematic representation in the absence or presence of cystine.

The interaction between GNPs and cystine was studied in an aqueous solution. The GNPs used in these experiments exhibited a uniform monodisperse size distribution with an average diameter of 15 nm (Figure S1). Transmission electron microscopy (TEM) images revealed that in the absence of cystine, GNPs maintained a monodispersed structure (Figure 1A). However, after adding cystine, a distinct morphological change occurred, with the GNPs aggregating into dense, clumpy structures. Notably, the nanoparticles clustered at regular intervals while retaining their individual shapes, forming a bridge-like connection between the particles (Figure 1B, middle). High-magnification TEM images further revealed an amorphous layer enveloping the GNPs, which resembled a stocking structure (Figure 1B). This type of interface is characteristic of organic material, as previously reported in the literature [32,33].

To confirm that the amorphous layer was composed of cystine, energy-dispersive spectroscopy (EDS) mapping was performed. The GNPs incubated with cystine showed a significant increase in nitrogen distribution around the nanoparticles (Figure 1C,D). Quantitative analysis indicated that nitrogen, originating from cystine, was approximately 13.5 times more concentrated around the GNPs compared to the GNP-only sample (Figure 1E,H). These results, illustrated in Figure 1F,G, demonstrate that cystine effectively interacts with the surface of GNPs, facilitating nanoparticle aggregation while preserving their individual structural integrity. Research suggests that these interactions occur through the sulfur and amine groups in cystine, as documented in earlier studies [19,20,21].

### 3.2. Cystine-Induced Aggregation of GNPs: Visual and Spectral Transition

**Figure 2:**
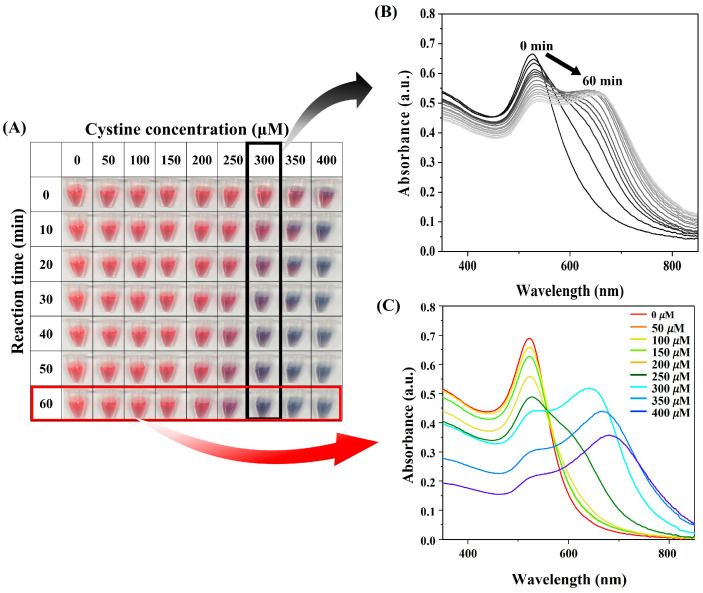
(**A**) Time kinetics visual observation of GNPs at different cystine concentrations; (**B**) UV-vis spectra of GNPs with cystine concentration of 300 µM over time; (**C**) UV-vis spectra at varying cystine concentrations after 1 h.

The aggregation of GNPs is typically associated with a color change from red to blue, accompanied by a corresponding shift in the ultraviolet–visible (UV-vis) absorption spectrum [34,35]. Initially, GNPs without cystine exhibited a red color and an absorption peak at 525 nm. Upon the addition of cystine, the solution gradually transitioned to blue over time, with the speed and intensity of the color change depending on cystine concentration and reaction time (Figure 2A). At low cystine concentrations, the red color persisted even after 60 min, suggesting that a minimum threshold concentration of cystine is required for significant aggregation.

At a cystine concentration of 300 µM, the solution exhibited a clear blue color and a significant red shift in the UV-vis absorption spectrum (Figure 2B). The observed red shift, coupled with a decrease in the surface plasmon resonance (SPR) intensity at 525 nm, correlated with the extent of GNP aggregation. Furthermore, the rate and extent of aggregation increased with higher cystine concentrations, as shown in Figure 2C. These findings support the hypothesis that cystine adsorbs onto the surface of GNPs, inducing interparticle cross-linking and aggregation. The red-to-blue color transition and the associated UV-vis spectral changes provide a reliable and quantitative method for monitoring this interaction. Furthermore, to confirm selectivity, we conducted experiments using an artificial tear solution containing common tear components, such as salts (e.g., NaCl), glucose, and urea, to assess potential interference. As demonstrated in Figure S2, no significant changes in the UV-vis spectra of GNPs were observed in the absence of cystine. However, in the presence of cystine, a noticeable red shift in the UV-vis peak occurred, demonstrating the sensor’s responsiveness.

### 3.3. Effect of Temperature on the Reaction

**Figure 3:**
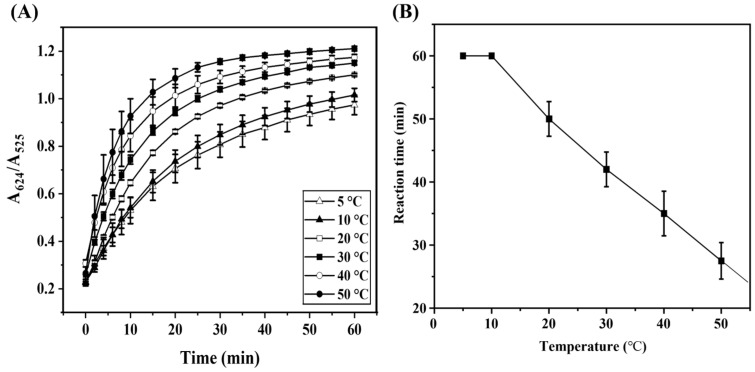
(**A**) Time kinetics plot of the absorbance ratio (A624/A525) at various temperatures (5–50 °C), where A625 is the absorption value at 624 nm and A525 is that at 525 nm; (**B**) plot of reaction completion time, determined at the point where the rate of absorbance change becomes negligible.

The influence of temperature on the GNP–cystine interaction was examined by monitoring the ratio of absorbance at 624 nm to 525 nm (Figure 3A). As expected, the reaction exhibited a red shift in the UV absorption spectra over time, and the absorbance ratio increased as the reaction progressed, reaching a saturation point at various temperatures. The time to reach saturation was consistent across all temperatures, with 1 h being sufficient for reaction completion (Figure 3B). The reaction rate constant (k) at each temperature was determined using the natural logarithmic slope of the decrease in absorbance at 525 nm, which corresponds to the concentration of reactants. Using the Arrhenius equation [36], the activation energy of the reaction was estimated to be 21.28 kJ/mol, indicating that temperature plays a significant role in the reaction kinetics.

### 3.4. Thermodynamic Insights into the Interaction Mechanisms

**Figure 4:**
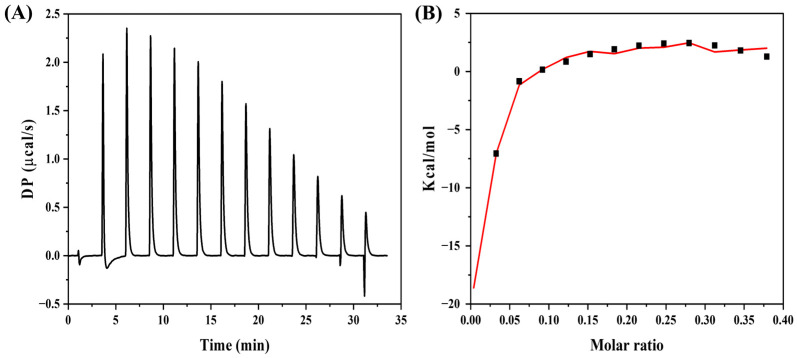
Isothermal titration calorimetry results: (**A**) raw heat data of cystine binding to GNPs; (**B**) integrated heat plots fitted using a sequential binding model.

To further understand the interaction between GNPs and cystine, isothermal titration calorimetry (ITC) was employed to determine the thermodynamic parameters governing this interaction. The raw heat data for cystine binding to GNPs are shown in Figure 4, with the resulting thermodynamic parameters summarized in Table 1. A sequential binding model with three binding sites was used to fit the data, representing the following key interactions: (1) the binding of cystine’s sulfur group to the GNPs, (2) the binding of the amine group to GNPs, and (3) the dissociation of citrate, the stabilizing agent on GNPs, upon cystine binding [19,20,21,37,38,39].

The data show that both enthalpy (ΔH) and free energy (ΔG) changes were negative (ΔH < 0, ΔG < 0), indicating that the binding of cystine to GNPs is an exothermic and spontaneous process. The dissociation constants (Kd) for each interaction site were used to compare the binding strength of cystine to GNPs. The lower Kd and larger enthalpy change observed in Set 1 suggest a stronger interaction compared to Set 2. Set 3 corresponds to the endothermic process involving the dissociation of citrate, which was confirmed by positive changes in both enthalpy and free energy (ΔH > 0, ΔG > 0). These findings provide a deeper understanding of the interaction mechanisms between GNPs and cystine, with multiple binding modes, including sulfur bonding (Set 1), amine bonding (Set 2), and citrate dissociation (Set 3).

### 3.5. Investigation of Cystine Removal by GNPs

To evaluate the ability of GNPs to remove cystine, ultra-performance liquid chromatography–mass spectrometry (UPLC-MS) was employed. The calibration curve for cystine concentration showed a linear relationship, demonstrating the suitability of this technique for quantitative analysis (Figure S3).

**Figure 5:**
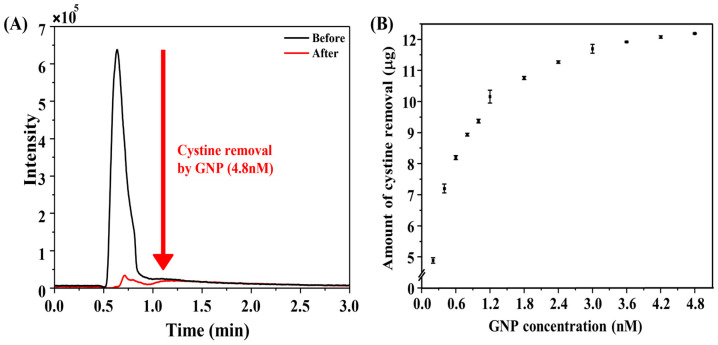
(**A**) UPLC-MS chromatograms showing cystine before and after reaction with GNPs; (**B**) quantification of cystine removal at varying GNP concentrations.

The chromatograms revealed a significant reduction in cystine concentration after reaction with GNPs, indicating effective cystine removal (Figure 5A). At a GNP concentration of 1.2 nM, cystine uptake exceeded 10 µg, suggesting substantial removal. However, at higher cystine concentrations, the removal amount reached saturation (Figure 5B). These results highlight that even small amounts of GNPs are capable of significantly lowering cystine levels, suggesting therapeutic potential for ocular cystinosis.

### 3.6. Theranostic GNP-CL for Ocular Cystinosis

**Figure 6:**
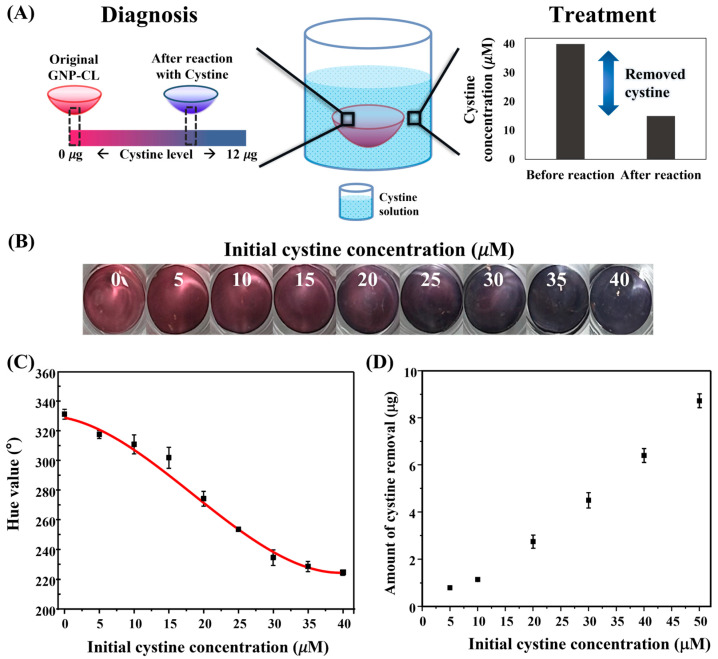
(**A**) Schematic of theranostic GNP-CL; (**B**) images of GNP-CL after reaction with cystine at various concentrations; (**C**) quantitative analysis of cystine concentration based on hue values; (**D**) cystine removal at varying concentrations.

The potential application of GNP-loaded contact lenses (GNP-CL) for theranostic purposes in ocular cystinosis was investigated, as illustrated in Figure 6A.

The GNP-CL exhibited a red color similar to the GNP solution (Figure S4A), with approximately 1.2 nM of GNPs loaded, which corresponds to the optimal concentration for cystine removal. No significant differences in contact angle or water content were observed between the GNP-CL and control lenses, indicating that the properties of the lens were not altered by the GNP loading (Figure S4B,C). The stability of GNPs within the contact lens has been demonstrated in a previous study [41]. To further verify this stability, we analyzed the UV-vis spectra of the solution after immersing GNP-CL in distilled water and PBS (pH 7.5) for up to 8 h. The spectra showed no significant absorbance at 525 nm, the characteristic wavelength of GNPs (Figure S5), indicating that GNPs remain stable within the lens matrix and are unlikely to leach into the ocular environment while wearing GNP-CLs. Moreover, the GNP-CL exhibited an average light transmittance of 60%, exceeding the transmittance range of conventional sunglasses (20–30%). This suggests minimal interference with the field of view (Figure S6).

After reaction, GNP-CLs demonstrated a color change from red to blue in response to increasing cystine concentrations (Figure 6B). GNP-CLs were quantitatively analyzed using hue values. The correlation between hue shift and cystine concentration was well fitted by a cubic regression model with a correlation coefficient of 0.99 (Figure 6C), providing a reliable method for cystine monitoring. The cystine concentration in the tears of healthy individuals is known to be about 1 μM [42], which is unlikely to induce a color change in the GNP-CL. Therefore, the GNP-CL is expected to function as a diagnostic tool, specifically for patients with cystinosis. Additionally, the remaining cystine in the solution was quantified using UPLC-MS. The amount of cystine removed was determined by analyzing the difference between before and after the reaction with GNP-CL, as shown in Figure 6D. This analysis confirmed that the GNP-CLs effectively removed cystine, with the amount of cystine removed positively correlating with the initial cystine concentration. Compared to conventional treatments such as cysteamine that indicated efficacy of 27–150 μg over an 8 h period, the GNP-CL has demonstrated the ability to remove up to 9 μg of cystine per hour, eliminating considerable variability due to inherent losses associated with topical formulations. A previous study has reported that the amount of cystine in is about 9 μg/h [6]. This aligns with the 9 μg per hour removal capacity demonstrated by our GNP-CL, indicating its potential to achieve sufficient treatment efficacy. Also, gold nanoparticles within the GNP-CL were incorporated after confirming their uniform size and shape. This allows for precise quantification and/or control of GNP loading, which provides optimizing therapeutic effects. These results suggest that GNP-CLs could serve as a non-invasive real-time tool for cystine monitoring, with potential therapeutic applications for ocular cystinosis by reducing cystine levels and preventing crystal formation.

## 4. Conclusions

In this study, we demonstrated the potential of a theranostic contact lens for managing ocular cystinosis, driven by the interaction between GNPs and cystine. In aqueous solutions, cystine’s sulfur and amine groups were found to bind to the surface of GNPs. This interaction helped stabilize the nanoparticles, with cystine acting as a bridge, thereby maintaining the integrity of the GNPs and facilitating their aggregation. This resulted in a visible color change (from red to blue) and a shift in the UV-vis absorption peak (525 to 624 nm), both indicative of GNP aggregation. When applied to the GNP-CL system, these properties allowed the lens to effectively bind cystine, facilitating the real-time visual monitoring of cystine levels in the eye. The maximum cystine removal observed from the GNP-CL was approximately 9 µg, which was comparable to the cystine removal by cysteamine, the standard treatment for ocular cystinosis. These findings suggest that GNP-CLs could serve as a potential theranostic tool for ocular cystinosis, offering both diagnostic and therapeutic capabilities.

Based on the results of this study, we propose that GNP-CLs could provide several key benefits for patients with ocular cystinosis. 1. Objective and quantitative evaluation: the colorimetric method employed by GNP-CL allows for an accurate and objective quantification of cystine levels, avoiding the subjective and labor-intensive nature of the traditional diagnostic method. 2. Patient-driven monitoring: the visual changes in the lens provide an intuitive tool for patients to actively monitor their condition, empowering them to take part in managing their disease. 3. Prevention of overdose: by continuously monitoring cystine levels, GNP-CLs could help adjust cysteamine doses, reducing the risks associated with overdose or underdose and minimizing side effects such as irritation. 4. Improved therapeutic outcomes: GNP-CLs may reduce reliance on cysteamine, addressing challenges such as storage limitations and the discomfort associated with its use. Moreover, it could potentially contribute to better treatment outcomes through more consistent monitoring. This work lays the foundation for the development of GNP-CLs as a theranostic wearable device, combining diagnostic monitoring with therapeutic effects. Future studies should focus on improving the reactivity of GNP-CLs through nanoparticle functionalization and expanding its application to other ocular diseases or conditions requiring similar monitoring. Overall, the GNP-CL has the potential to revolutionize the management of ocular cystinosis by offering a simple, non-invasive, and patient-centered solution. Further research could enhance its clinical applicability, making it a viable tool for improving both the quality of life and clinical outcomes for cystinosis patients.

## Figures and Tables

**Table 1:** Binding constants and thermodynamic parameters derived from ITC data as cystine were titrated into the GNP solution. Set 1: binding of cystine’s sulfur group; Set 2: bonding of cystine’s amine group; Set 3: dissociation of citrate, the GNP stabilizer, to GNPs.

Set Type	Kd (nM)	ΔH (kcal/mol)	ΔG (kcal/mol) ^1^	−TΔS (kcal/mol)
Set 1	12.2	−38.9	−67	28.1
Set 2	44.8	−19.6	−29.15	9.55
Set 3	3.14	42.1	95.8	−53.7

^1^ ΔG was calculated using the equation ΔG = ΔH − TΔS [40].

## Data Availability

The data is contained within the article and Supplementary Materials.

## Supplementary Materials

The following supporting information can be downloaded at: https://www.mdpi.com/article/10.3390/bios15010016/s1, Figure S1: (A) UV-vis spectra of GNP showing a characteristic absorption peak at around 525 nm.; (B) dynamic light scattering results; and (C) TEM image exhibiting a monodisperse uniform size distribution with an average diameter of 15 nm; Figure S2: UV-vis spectra of GNP in the artificial tear (A) with the absence of cystine and (B) the presence of cystine; Figure S3: (A) UPLC-MS chromatograms and (B) calibration curve of cystine solution. The retention time was consistently recorded at 0.63 min, with the standard curve for peak area yielding an *R*2 value of approximately 0.99, confirming the reliability of quantification; Figure S4: Comparison of the characteristics between unloaded CLs (control) and GNP-CLs; (A) color image, (B) contact angle values, and (C) water content measurement results. The GNP-CLs exhibited contact angles below 90° and a water content of approximately 61%, with no significant differences compared to control lenses; Figure S5: UV-vis spectra of measuring (A) distilled water solution and (B) PBS solution in which the GNP-CL was immersed for up to 8 h; Figure S6: Average light transmittance graph of GNP-CLs.

## References

[1] Gahl W.A., Thoene J.G., Schneider J.A. (2002). Cystinosis. N. Engl. J. Med..

[2] Gahl W.A., Thoene J.G., Schneider J.A. (2001). Cystinosis: A disorder of lysosomal membrane transport. Metab. Mol. Bases Inherit. Dis..

[3] Town M., Jean G., Cherqui S., Attard M., Forestier L., Whitmore S.A., Callen D.F., Gribouval O., Broyer M., Bates G.P. (1998). A novel gene encoding an integral membrane protein is mutated in nephropathic cystinosis. Nat. Genet..

[4] Tsilou E., Zhou M., Gahl W., Sieving P.C., Chan C.-C. (2007). Ophthalmic Manifestations and Histopathology of Infantile Nephropathic Cystinosis: Report of a Case and Review of the Literature. Surv. Ophthalmol..

[5] Kaiser-Kupfer M.I., Caruso R.C., Minkler D.S., Gahl W.A. (1986). Long-term Ocular Manifestations in Nephropathic Cystinosis. Arch. Ophthalmol..

[6] Liu Z., Kompella U.B., Chauhan A. (2021). Gold nanoparticle synthesis in contact lenses for drug-less ocular cystinosis treatment. Eur. J. Pharm. Biopharm..

[7] Kowalczyk M., Toro M.D., Rejdak R., Załuska W., Gagliano C., Sikora P. (2020). Ophthalmic Evaluation of Diagnosed Cases of Eye Cystinosis: A Tertiary Care Center’s Experience. Diagnostics.

[8] Pinxten A.-M., Hua M.-T., Simpson J., Hohenfellner K., Levtchenko E., Casteels I. (2017). Clinical Practice: A Proposed Standardized Ophthalmological Assessment for Patients with Cystinosis. Ophthalmol. Ther..

[9] Gahl W.A., Tietze F., Butler J.D., Schulman J.D. (1985). Cysteamine depletes cystinotic leucocyte granular fractions of cystine by the mechanism of disulphide interchange. Biochem. J..

[10] Hector E., Cairns D., Wall G.M. (2022). Evaluation of NACA and diNACA in human cystinosis fibroblast cell cultures as potential treatments for cystinosis. Orphanet J. Rare Dis..

[11] Gulsen D., Chauhan A. (2004). Ophthalmic Drug Delivery through Contact Lenses. Investig. Opthalmol. Vis. Sci..

[12] Hsu K.-H., Fentzke R.C., Chauhan A. (2013). Feasibility of corneal drug delivery of cysteamine using vitamin E modified silicone hydrogel contact lenses. Eur. J. Pharm. Biopharm..

[13] Gahl W.A., Kuehl E.M., Iwata F., Lindblad A., Kaiser-Kupfer M.I. (2000). Corneal Crystals in Nephropathic Cystinosis: Natural History and Treatment with Cysteamine Eyedrops. Mol. Genet. Metab..

[14] Ryu J.H., Koo H., Sun I.C., Yuk S.H., Choi K., Kim K., Kwon I.C. (2012). Tumor-targeting multi-functional nanoparticles for theragnosis: New paradigm for cancer therapy. Adv. Drug Deliv. Rev..

[15] Zhou H., Tang D., Yu Y., Zhang L., Wang B., Karges J., Xiao H. (2023). Theranostic imaging and multimodal photodynamic therapy and immunotherapy using the mTOR signaling pathway. Nat. Commun..

[16] Shi Z., Lu Y., Shen S., Xu Y., Shu C., Wu Y., Lv J., Li X., Yan Z., An Z. (2022). Wearable battery-free theranostic dental patch for wireless intraoral sensing and drug delivery. NPJ Flex. Electron..

[17] Han F., Li J., Xiao P., Yang Y., Liu H., Wei Z., He Y., Xu F. (2024). Wearable smart contact lenses: A critical comparison of three physiological signals outputs for health monitoring. Biosens. Bioelectron..

[18] Liu X., Ye Y., Ge Y., Qu J., Liedberg B., Zhang Q., Wang Y. (2024). Smart Contact Lenses for Healthcare Monitoring and Therapy. ACS Nano.

[19] López-Tobar E., Hernández B., Ghomi M., Sanchez-Cortes S. (2013). Stability of the Disulfide Bond in Cystine Adsorbed on Silver and Gold Nanoparticles As Evidenced by SERS Data. J. Phys. Chem. C.

[20] Yao G., Huang Q. (2018). DFT and SERS Study of l-Cysteine Adsorption on the Surface of Gold Nanoparticles. J. Phys. Chem. C.

[21] Ben Haddada M., Blanchard J., Casale S., Krafft J.-M., Vallée A., Méthivier C., Boujday S. (2013). Optimizing the immobilization of gold nanoparticles on functionalized silicon surfaces: Amine- vs thiol-terminated silane. Gold Bull..

[22] Montaño-Priede J.L., Sanromán-Iglesias M., Zabala N., Grzelczak M., Aizpurua J. (2023). Robust Rules for Optimal Colorimetric Sensing Based on Gold Nanoparticle Aggregation. ACS Sens..

[23] Saha K., Agasti S.S., Kim C., Li X., Rotello V.M. (2012). Gold Nanoparticles in Chemical and Biological Sensing. Chem. Rev..

[24] Han G., Ghosh P., Rotello V.M. (2007). Functionalized Gold Nanoparticles for Drug Delivery. Nanomedicine.

[25] Abdelhamid H.N., Badr G. (2021). Nanobiotechnology as a platform for the diagnosis of COVID-19: A review. Nanotechnol. Environ. Eng..

[26] Dixit S., Novak T., Miller K., Zhu Y., Kenney M.E., Broome A.-M. (2015). Transferrin receptor-targeted theranostic gold nanoparticles for photosensitizer delivery in brain tumors. Nanoscale.

[27] Turkevich J., Stevenson P.C., Hillier J. (1951). A study of the nucleation and growth processes in the synthesis of colloidal gold. Discuss. Faraday Soc..

[28] Liu X., Atwater M., Wang J., Huo Q. (2007). Extinction coefficient of gold nanoparticles with different sizes and different capping ligands. Colloids Surf. B Biointerfaces.

[29] Qi X., Zhang H., Li Y., Zhang X., Ma H., Zhang L. (2022). Nonfouling and Antibacterial Zwitterionic Contact Lenses Loaded with Heme-Mimetic Gallium Porphyrin for Treating Keratitis. Langmuir.

[30] Tan Y., Guo M. (2013). Using surface free energy method to study the cohesion and adhesion of asphalt mastic. Constr. Build. Mater..

[31] Canbay E., Sezer E.D., Uçar S.K., Çoker M., Sözmen E.Y. (2020). LC-MS/MS measurement of leukocyte cystine; effect of preanalytic factors. Talanta.

[32] El Shafey A.M. (2020). Green synthesis of metal and metal oxide nanoparticles from plant leaf extracts and their applications: A review. Green Process. Synth..

[33] Kim M., Noh H. (2022). Study on Colloidal Stability of Gold Nanoparticles Modified with Sugar Molecules. Polym. Korea.

[34] Pu W., Zhao H., Huang C., Wu L., Xu D. (2013). Visual detection of arginine based on the unique guanidino group-induced aggregation of gold nanoparticles. Anal. Chim. Acta.

[35] Holme M.N., Rana S., Barriga H.M.G., Kauscher U., Brooks N.J., Stevens M.M. (2018). A Robust Liposomal Platform for Direct Colorimetric Detection of Sphingomyelinase Enzyme and Inhibitors. ACS Nano.

[36] Zheng J., Png Z.M., Ng S.H., Tham G.X., Ye E., Goh S.S., Loh X.J., Li Z. (2021). Vitrimers: Current research trends and their emerging applications. Mater. Today.

[37] Al-Johani H., Abou-Hamad E., Jedidi A., Widdifield C.M., Viger-Gravel J., Sangaru S.S., Gajan D., Anjum D.H., Ould-Chikh S., Hedhili M.N. (2017). The structure and binding mode of citrate in the stabilization of gold nanoparticles. Nat. Chem..

[38] Rao X., Tatoulian M., Guyon C., Ognier S., Chu C., Hassan A.A. (2019). A Comparison Study of Functional Groups (Amine vs. Thiol) for Immobilizing AuNPs on Zeolite Surface. Nanomaterials.

[39] Stobiecka M., Deeb J., Hepel M. (2010). Ligand exchange effects in gold nanoparticle assembly induced by oxidative stress biomarkers: Homocysteine and cysteine. Biophys. Chem..

[40] Li M., Zhang X., Li S., Shao X., Chen H., Lv L., Huang X. (2021). Probing protein dissociation from gold nanoparticles and the influence of temperature from the protein corona formation mechanism. RSC Adv..

[41] Salih A.E., Elsherif M., Alam F., Yetisen A.K., Butt H. (2021). Gold Nanocomposite Contact Lenses for Color Blindness Management. ACS Nano.

[42] Nakatsukasa M., Sotozono C., Shimbo K., Ono N., Miyano H., Okano A., Hamuro J., Kinoshita S. (2011). Amino Acid Profiles in Human Tear Fluids Analyzed by High-Performance Liquid Chromatography and Electrospray Ionization Tandem Mass Spectrometry. Arch. Ophthalmol..

