# Supplementary material for: Theranostic Contact Lens for Ocular Cystinosis Utilizing Gold Nanoparticles
Source: Biosensors (Basel). 2025 Jan 3;15(1):16. doi: 10.3390/bios15010016 (PMC11764065; doi:10.3390/bios15010016)
Supplement: Supplementary file 1 [file biosensors-15-00016-s001.zip › biosensors-3367622-supplementary.pdf]

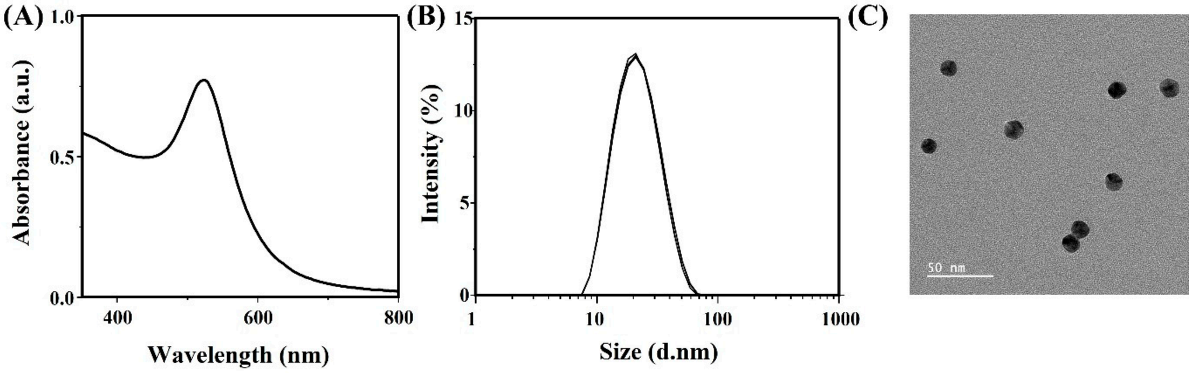

Figure S1. (A) UV-vis spectra of GNP showed a characteristic absorption peak at around 525 nm.; (B) Dynamic light scattering results and (C) TEM image exhibited a monodisperse, uniform size distribution with an average diameter of 15 nm.

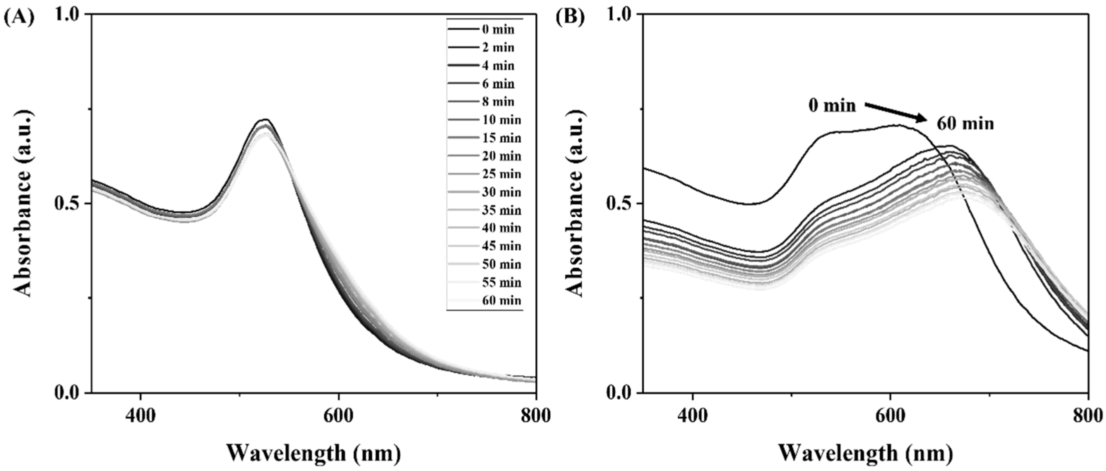

Figure S2: UV-vis spectra of GNP in the artificial tear (A) with the absence of cystine and (B) the presence of cystine.

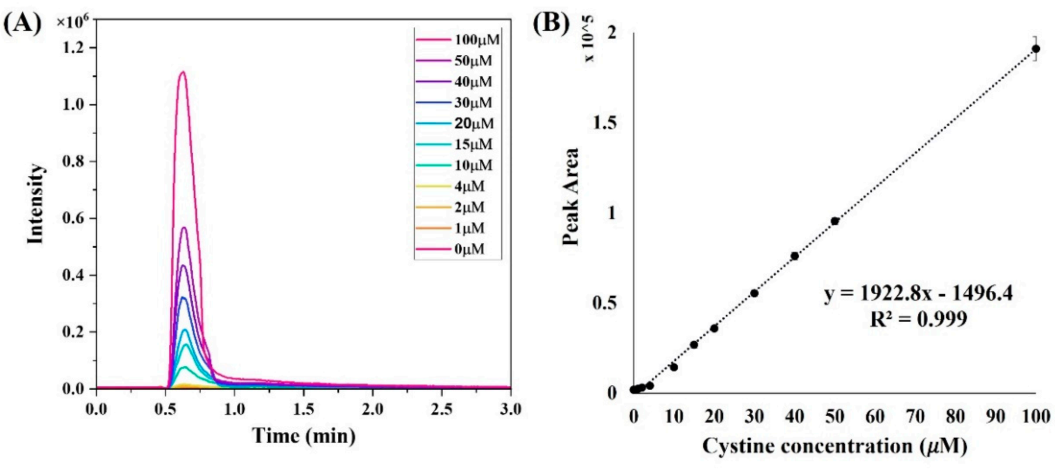

Figure S3: (A) UPLC-MS chromatograms and (B) calibration curve of cystine solution.; The retention time was consistently recorded at 0.63 minutes, with the standard curve for peak area yielding an $R2$ value of approximately 0.99, confirming the reliability of quantification.

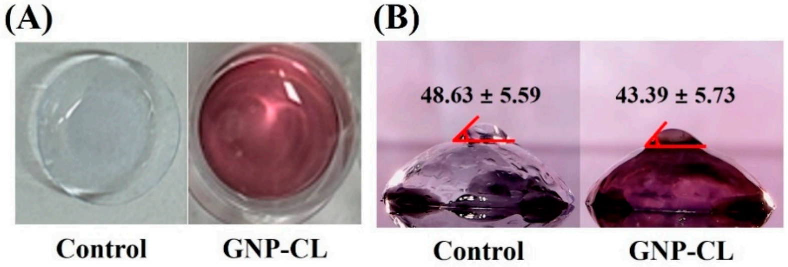

(C)

Sample	Wet weight (g)	Dry weight (g)	Water content (%)
Control	0.0359±0.0022	0.0143±0.0001	60.1342±2.4327
GNP-CL	0.0373±0.0018	0.0144±0.0001	61.3281±1.7597

Figure S4: Comparison of the characteristics between unloaded CL (control) and GNP-CL; (A) color image, (B) contact angle values and (C) water content measurement results.; The GNP-CLs exhibited contact angles below 90° and a water content of approximately 61 %, with no significant differences compared to control lenses.

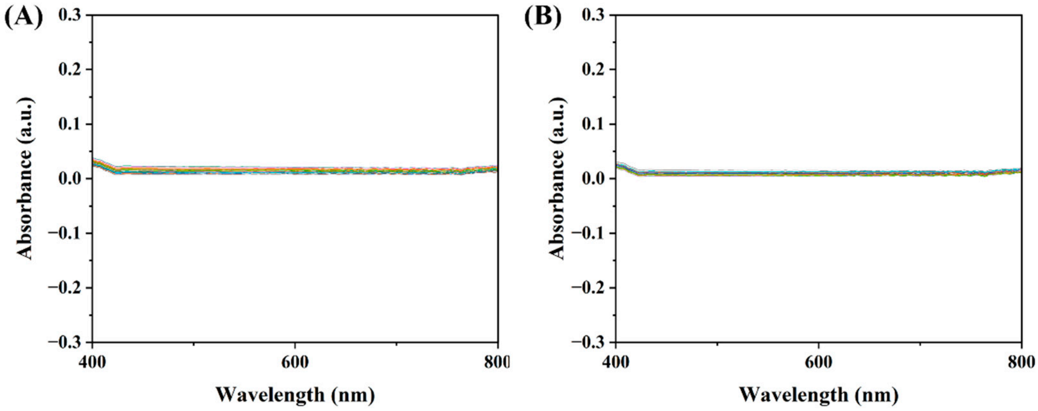

Figure S5: UV-vis spectra of measuring (A) distilled water solution and (B) PBS solution in which the GNP-CL was immersed for up to 8 hours.

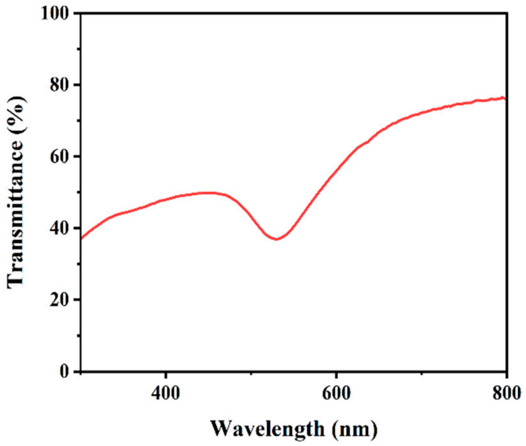

Figure S6. Average light transmittance graph of GNP-CL.
